# A Digital Health Intervention for Weight Management for Latino Families Living in Rural Communities: Perspectives and Lessons Learned During Development

**DOI:** 10.2196/20679

**Published:** 2020-08-20

**Authors:** Zenong Yin, Vanessa L Errisuriz, Martin Evans, Devasena Inupakutika, Sahak Kaghyan, Shiyu Li, Laura Esparza, David Akopian, Deborah Parra-Medina

**Affiliations:** 1 Department of Kinesiology, Health, and Nutrition The University of Texas at San Antonio San Antonio, TX United States; 2 Latino Research Institute The University of Texas at Austin Austin, TX United States; 3 Department of Electrical and Computer Engineering The University of Texas at San Antonio San Antonio, TX United States

**Keywords:** mhealth, digital intervention, Latino families, rural population, weight, self-management, diet, lifestyle, chronic disease

## Abstract

Rural residents face numerous challenges in accessing quality health care for management of chronic diseases (eg, obesity, diabetes), including scarcity of health care services and insufficient public transport. Digital health interventions, which include modalities such as internet, smartphones, and monitoring sensors, may help increase rural residents’ access to health care. While digital health interventions have become an increasingly popular intervention strategy to address obesity, research examining the use of technological tools for obesity management among rural Latino populations is limited. In this paper, we share our experience developing a culturally tailored, interactive health intervention using digital technologies for a family-oriented, weight management program in a rural, primarily Latino community. We describe the formative research that guided the development of the intervention, discuss the process of developing the intervention technologies including issues of privacy and data security, examine the results of a pilot study, and share lessons learned. Our experience can help others design user-centered digital health interventions to engage underserved populations in the uptake of healthy lifestyle and disease management skills.

## Introduction

The ownership of cellular phones, smartphones, and tablet computers has reached historic heights across all US population groups in recent years [1]. In 2018, at least 75% of Blacks/African Americans, Latinos, and non-Latino Whites and 65% of rural residents owned a smartphone. Advances in information technology (IT), along with the diminishing digital divide among population groups, have increased the use of digital technologies in health promotion and disease management [2]. As a result, individuals from low-income and racial/minority backgrounds, who traditionally had less access to health information, are now able to more easily access health information via Internet with mobile devices [3,4]. Diffusion of digital technologies has also popularized the use of digital health interventions to reach rural and resource-poor communities with limited access to traditional tools and programs for health promotion and disease prevention and management [5,6].

In the realm of digital health, Internet- and mobile-based IT platforms (eg, SMS text messages, websites, social media, wearable technology) have been used to store, manage, and transmit data using hardware, software, and networks for delivering health care services and health promotion programs [7]. Mobile health (mHealth) technology is an important subset of digital health using mobile phones, tablets, apps, and other wireless technologies. Compared with traditional face-to-face health care delivery, digital health interventions have demonstrated great promise in enhancing program reach and participant engagement, and achieving similar effectiveness in health-related outcomes. [7,8]. However, mHealth interventions often limit the number of behavior change techniques (BCTs) and rarely offer problem solving, social support, and didactic education [9,10]. Engagement of historically underserved populations in digital health interventions is influenced by a variety of factors, such as the extent to which program content is tailored and formatted, the alignment between devices used for program delivery and population needs [6], and the appropriateness of program context [11]. Additional research indicates that personalization of content, contact with program users [12], and preference of digital devices to receive content are also important indicators of program engagement [13]. For example, Latino users are more likely to stop using health-related mobile apps due to access issues (eg, rely primarily on mobile phones versus tablets or computers), lack of interest in the content, and high cost [14,15]. The development of digital health interventions must address these barriers to increase participation among underserved populations.

Healthy Frio is a translation study of the Y-Living Program (Y-Living), an evidence-based, family-focused intervention designed for urban Latino families (Trial Registration: ClinicalTrials.gov NCT03186885) [16]. The translation of Y-Living is a two-phased study guided by the principles of community-based participatory research [17]. The first phase of the study engaged community partners in formative research to adapt Y-Living for rural Latino families. Formative research included (1) a pilot of an in-person (IP) group program at a community center, and (2) a pilot of a home-based program remotely delivered by digital technologies. While we designed both forms of program delivery to address unique social, cultural, and environmental factors facing rural Latino families, the latter takes advantage of digital technologies to increase program availability, accessibility, and engagement. More specifically, the objectives of using remote delivery were to (1) increase access to evidence-based health education content and resources; (2) address learning needs with content design; (3) address the need for individualized support, and (4) support behavior change with wearable technologies grounded in behavior change theory. The second phase of the study is a 12-month randomized controlled trial (RCT) that tests the comparative effectiveness of the Y-Living IP and remote technology (RT) interventions on weight loss and energy balance behaviors among overweight and obese rural Latino adults relative to a control group. Following baseline assessments and randomization, intervention participants receive a 12-week IP or RT program, whereas control participants receive 1 brief health education session and educational materials. Study outcomes will be assessed again immediately following the intervention at 3 months, 6 months after intervention, and 12-month follow-up.

In this paper, we describe the process undertaken by a multidisciplinary research team to develop the RT intervention for the Healthy Frio study. Key elements of the process include the formative research that guided the development of the RT intervention, the selection and integration of digital technologies [18], and a pilot study. Our experience and lessons learned can help inform decisions other researchers may face when designing user-centered digital health interventions to engage underserved populations in the uptake of healthy lifestyle and disease management skills.

## Overview of the Development Process

**Figure 1:**
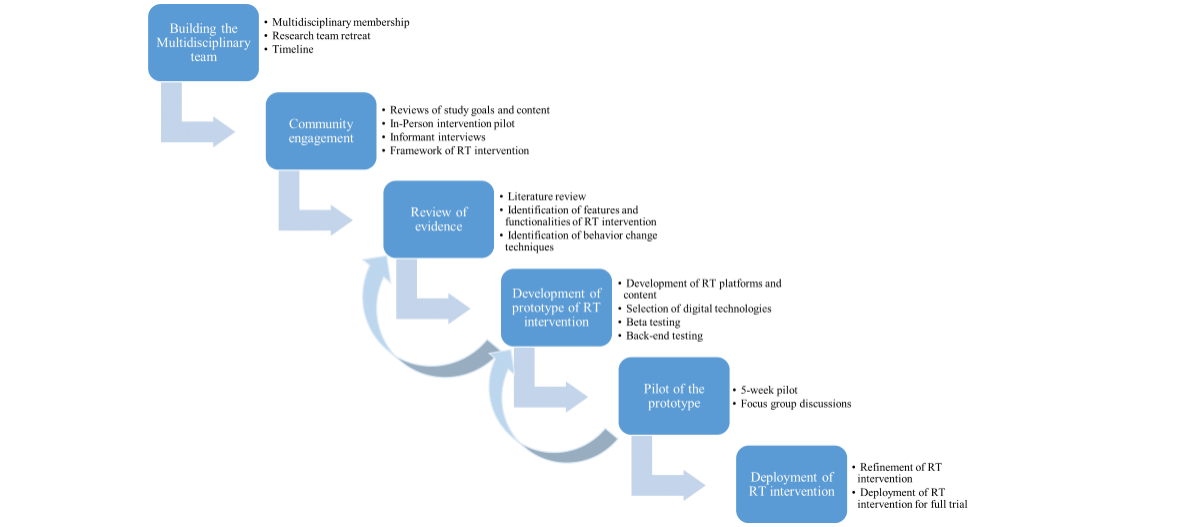
Healthy Frio remote technology (RT) intervention development process.

The development of the RT intervention took place over 16 months, from the formation of the research team to the deployment of the RT intervention for the full RCT. The process included several steps: (1) formulating the research team, (2) engaging community partners and residents to adapt the Y-Living for rural Latino families, (3) reviewing the evidence on use of digital technologies for behavior change, (4) developing a prototype of the RT intervention in parallel with IP intervention, and (5) conducting a pilot test of the prototype. Figure 1 shows the development process of Healthy Frio RT intervention.

### Formulation of RT Development Team

A multidisciplinary team including researchers with expertise in computer engineering, database and instructional design, and public and behavioral health, and primary care practitioners (community/family medicine and nursing) developed the RT intervention. The instructional design specialist was designated as the coordinator. The technology modules and components integrated as a part of the RT intervention required development of necessary links, incorporation of application program interfaces (APIs), service interfaces, dashboards, and testing of interoperability, compatibility, and robustness. Thus, digital health RT interventions demand the expertise of a professional team of computer engineers to develop and manage seamless functioning and efficient intervention delivery in collaboration with behavioral health researchers who understand behavior change processes. A team science approach was critical in the communication and collaboration between computer engineers and behavioral health researchers and the development and management of the multidisciplinary team [19]. Ultimately, this approach helps in scaling and disseminating effective interventions. During the first year of development, the RT team met biweekly via conference call to discuss and develop the intervention and then met monthly after the first year. Additionally, the team met IP for 2 full-day retreats over the 16-month development period.

### Community Engagement

Y-Living is a 12-week behavioral modification program grounded in the Social Cognitive Theory of behavior change to engage the whole family in lifestyle changes [20]. During this program, participants develop knowledge and skills to be physically active and eat healthy, set goals for changes, self-monitor their physical activity and diet, and create a supportive environment at home. Program components of Y-Living include semiweekly health education lessons; semiweekly group exercise sessions; self-monitoring of weight, physical activity, and diet; family wellness consultations; and special events. Trained Young Men’s Christian Association (YMCA) staff and volunteers deliver the program activities in YMCA facilities. The Y-Living program was designed for urban Latino families. To translate Y-Living into an efficacious program for rural Latino families, the Healthy Frio intervention team, including the YMCA wellness coordinator, conducted an extensive review of Y-Living program activities and sought feedback from community partners regarding feasibility of program activities for a rural environment. The Healthy Frio intervention team reviewed and updated health education lessons to reflect current, evidence-based recommendations for weight-loss, physical activity, and diet that addressed the needs of rural populations. Some lessons were omitted and replaced with new topics that more closely aligned with behavior change strategies identified in the Social Cognitive Theory and the Coventry, Aberdeen, and London-Refined taxonomy [21,22], such as goal-setting, self-monitoring, feedback, rewards, social support, coaching, problem solving, and action planning. A 5-week pilot with 5 parent–child dyads was used to test the feasibility of the adapted IP intervention in the community. Table 1 displays the Healthy Frio intervention components for both the IP and RT interventions and demonstrates program alignment. The intervention team also conducted key informant interviews with community stakeholders to gather information on access and availability to physical activity and healthy eating in the community, availability of digital technologies and supportive resources in the community, and level of health technology literacy. The intervention team reviewed program content and delivery approaches prior to the deployment of the RT intervention in the pilot study and the full trial.

**Table 1:** Description of and alignment between in-person and remote technology intervention.

Mediators	Strategies	In-person intervention	Remote technology intervention
Self-regulation	Initial wellness report	Provided at first wellness consultation. Includes height, weight, BMI, average PA^a^ minutes, and caloric intake recorded at baseline assessments. A YMCA^b^ wellness coach discusses with each participant and helps him/her set goals to increase PA and lower calorie intake, and set a target weight loss goal (5%) for 3-month assessment.	Provided at first wellness consultation. Includes height, weight, BMI, average PA minutes, and caloric intake recorded at baseline assessments Health educator discusses with each participant and helps him/her set goals to increase PA and lower calorie intake, and set a target weight loss goal (5%) for 3-month assessment.
Self-efficacy Perceived barriers Social support	Orientation session	Participants receive program materials (eg, program binder, self-monitoring logs) and information about the program schedule and goals.	Participant is provided, and given instructions on how to use, all equipment (eg, tablet, Fitbit), apps, timeline, program goals, and expectations.
Home environment Self-efficacy Self-regulation	Healthy living lessons	12 weeks of lessons at community center: 2 lessons per week.	12 weeks of online lessons delivered via tablet computers: 2 lessons per week.
Self-efficacy Perceived barriers	Cooking demonstrations	Brief cooking demonstration by YMCA staff following health education lessons.	Video recordings of cooking demonstrations conducted by YMCA staff.^c^
Home environment Self-efficacy Self-regulation Perceived barriers Social support	Physical activity sessions	Immediately following Y Living lessons, a 1-hour PA session led by YMCA wellness coach. Examples of activities include circuit training, Zumba, and games from CATCH^d^ curriculum. Children and adults are usually not separated.	PA sessions (for both children and adults) are included in online lessons using videos curated from YouTube, as well as 5 videos produced by Healthy Frio and YMCA wellness coaches. Separate videos are provided that are oriented toward adults and children.
Self-efficacy Self-regulation Perceived barriers	Self-monitoring	Paper and pencil log for PA and food intake. Weigh-ins during individual consultation.	Use of Fitbit, scale, apps, etc.
Self-efficacy Self-regulation	SMS text messaging	2 per week. Healthy Lifestyle tips for increasing PA and healthy eating.	Lesson reminders, health challenges, same healthy lifestyle tips as in-person, polling question regarding health challenges.
Social support Perceived barriers	Grocery store tour	Participants taken to a local grocery store where they are led on a tour and given tips for healthy shopping (eg, comparing labels, prices, and suggested routes through grocery store [perimeter first]).	Virtual grocery store tour lesson using video segments for each section of grocery store.
Home environment Social support	Wellness consultations	Small group discussions and coaching sessions.	Group video calls via Google Hangouts^e^ or Skype (weeks 2, 6, and 10).
Home environment Self-efficacy Social support	One-on-one meetings	3 individual consultations between YMCA staff and participants to review progress report (weeks 2, 5, and 8).	Biweekly phone calls with each family, and a midprogram progress report sent via email in week 7.

^a^PA: physical activity.

^b^YMCA: Young Men’s Christian Association.

^c^Cooking demos performed at Y-Living Program in San Antonio (not part of Healthy Frio Trial).

^d^CATCH: Coordinated Approach to Child Health.

^e^Google Hangouts used for Cohort 1. Skype will be used for remaining cohorts.

### State-of-the-Art Review and Integration of Research Literature

As the third step of the development process, the RT team conducted a review of the literature on the application of digital technologies in health promotion and disease management from the perspectives of health promotion practices and computer engineering. Furthermore, the team conducted a state-of-the-art review on modern health data collection and sharing approaches in the context of an emerging gig economy [23]. The goal was to understand the progress, best practices, and challenges in digital health intervention and provide guidance in designing a theory-driven RT intervention.

Application of digital health technologies has evolved from a single IT platform that focused on offering access to web-based intervention information to multiple platforms that extended exposure to the intervention in the context of the targeted behaviors. Research indicates that exposure is extended by (1) minimizing time-consuming data input and output activities, (2) automating delivery of task reminders and reinforced messages, (3) providing frequent and real-time feedback to intervention participants and staff, (4) offering the ability to tailor the intervention based on baseline information as well as ongoing changes of the behavior targets and the environment, and (5) leveraging the interactive capabilities of mHealth devices to incorporate BCTs [10,24]. By contrast, lack of structured social interactions and social support was a noted weakness of current digital technologies [23,24]. For example, studies have demonstrated that incorporating IP contact enhanced program compliance and participant engagement in mHealth interventions [25,26]. Early mHealth interventions have also been criticized for not using behavioral theories to inform the program design [7].

The RT team identified key features of successful digital health interventions such as two-way communication between the participants and intervention, social support from intervention staff and others, and some forms of competition [7,27,28]. For example, a systematic review found that successful interactive health IT systems for self-management in older, chronically ill, and underserved adults had a *completed feedback loop*. This feedback loop monitored the patient’s health status, provided the progress toward patient’s goals based on the monitoring, facilitated the adjustment of goals and management plans, and offered tailored advice and recommendations [29]. Using multiple IT platforms can increase intervention exposure and support for participants [27,28]. The RT team also identified important barriers influencing uptake of targeted health behaviors, including the program’s ability to address community interests and cultural context, perceived effectiveness of the intervention, difficulty accessing and navigating the systems (eg, interface design), reliability of the systems (eg, technical issues), ability to deliver the program using technologies familiar to participants, the extent to which intervention activities fit into daily routines, and timely and frequent contact with intervention staff [7,29,30].

Finally, contemporary health behavior theories and models have been developed primarily based on face-to-face interactions for the management of behavior changes and do not address the time-intensive and interactive nature and asynchronous and dynamic process of digital health interventions [31]. While the design of the content of digital health interventions has benefited from these theories and models, the design of delivery and feedback processes remains poorly guided. This weakness speaks about the lack of cooperation between computer system engineers and behavioral researchers [22,31] and the need to establish a model to guide the incorporation of BCTs into the content and functionality of digital health interventions [32].

Based on the results of the review, the RT team decided to adopt a user-centered system design [33,34] utilizing an iterative, participatory process to address learning goals, communication styles, and community context. This system design required participation from instructional designers, computer engineers, behavioral scientists, and end users (interventionist and participants). Applying the principles of a user-centered approach has contributed to increases in acceptance and usability of digital technology-based interventions [35,36]. From a computer engineering perspective, the RT team concluded that a blended approach, combining digital technologies with limited human high-touch, may be most efficacious to advance the field of digital health interventions while awaiting the next generation of artificial intelligence-based solutions to address weaknesses associated with inability to provide personalized social support [23,37].

### The Development of Prototype for Delivery of the RT Intervention

#### Delivery Platform for RT Intervention

**Figure 2:**
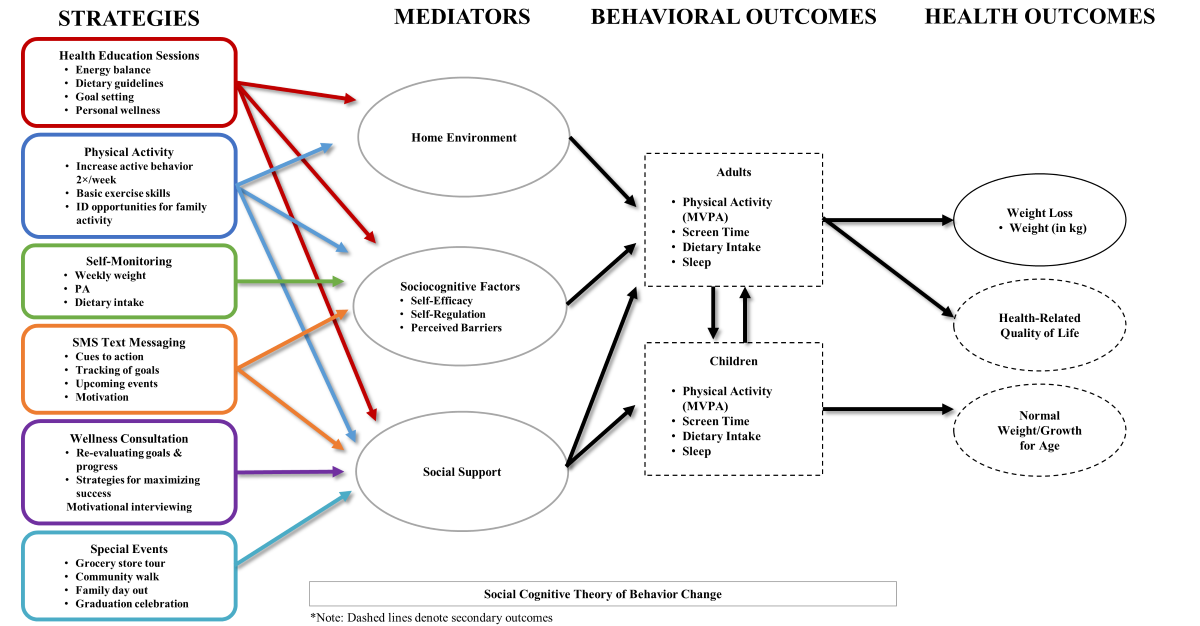
Healthy Frio remote technology intervention conceptual model. MVPA: moderate to vigorous physical activity; PA: physical activity.

The design of the Healthy Frio RT intervention was guided by a blended approach to create a program that is “both useful and easy to use, ie, at really serving the needs of the user,” following the principles of the persuasive design system (PDS) [38,39]. The RT team reached a consensus early on in the development process to implement the 4 features of PDS (primary task support, dialogue support, system credibility, and social support) by blending numerous IT platforms with the facilitation of a live lifestyle coach to take advantage of digital technologies and the desire of human touch in supporting behavior changes [37,40]. Table 2 presents the operationalization of PDS techniques in alignment with the Social Cognitive Theory in Healthy Frio RT intervention. There are compromises between the rigorousness of the program content and the limitations of technology due to costs and scalability [38]. Figure 2 depicts the conceptual framework of RT intervention development that integrates PDS technologies to address moderators of self-efficacy to influence targeted health behaviors and outcomes, based on the Social Cognitive Theory [20]. The combination of digital technologies and live coaching affords the most efficient support to engage the study participants with BCTs [22] and close the feedback loop while providing constant exposure to the intervention content [29]. The Healthy Frio intervention team reviewed the content and delivery approaches of the RT intervention prior to its deployment for the pilot study and the full trial.

**Table 2:** Implementation of features and techniques for designing the content and functionality of a persuasive design system.

PDS^a^ features	Operationalization of PDS techniques in Healthy Frio digital health intervention
Primary task support: Supporting and facilitating completion of the user’s main tasks	Guided goal-setting; self-monitoring of weight, physical activity, and diet; using simulation and rehearsal in online interactive health education; online health challenges to target specific behavior
Dialogue support: Providing support, guidance, and feedback to users by verbal or other form	Suggestion and reminders via SMS text messaging; praise and reinforcement via health app (Fitbit app), and live online coaching
System credibility support: Presenting program content as trustworthy and authoritative to users	Embedding spirituality in online health education; using respected and authoritative sources; connecting program content to local community; affiliating the program with trusted local entities
Social support: Motivating and engaging users by leveraging social influence	Using parent–child dyad; providing physical activity trackers and weight tracking accounts to dyads; weekly calls by lifestyle coach

^a^PDS: persuasive design system.

**Figure 3:**
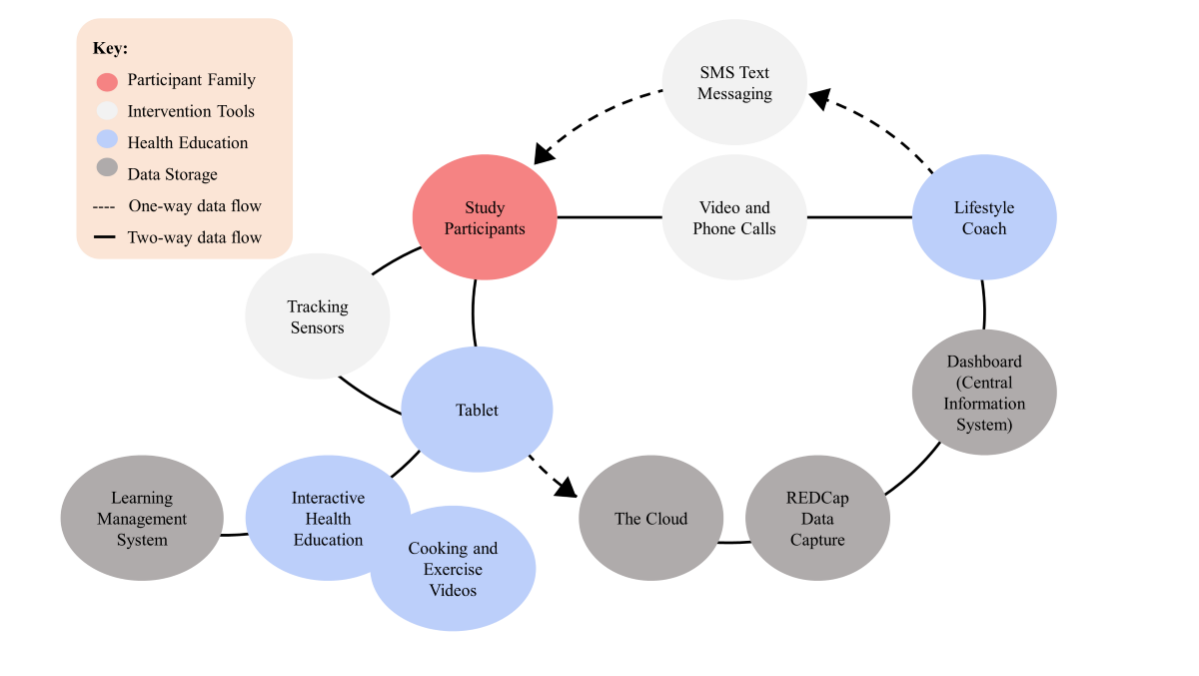
Healthy Frio remote technology intervention delivery platform.

The implementation of the RT intervention is accomplished by a multiple-system integrated platform that is designed to address 3 elements of behavior change for persuasive design in human–computer interactions: (1) motivating participants with goal setting, positive reinforcement, and social support; (2) increasing participant’s ability to adopt new behaviors by learning behavior change skills; and (3) providing cues to initiate and maintain new behavior via SMS text message reminders and feedback from digital technologies [38]. The platform included interactive, online health education lessons delivered via tablet; tracking sensors (activity tracker and Bluetooth weight scale) and apps to self-monitor activity, diet, and weight; social support and consultations in the form of phone calls, video chats, and motivation and support from automated SMS text messages; cooking and exercise videos; and a dashboard to allow the lifestyle coach to monitor and support participants (Figure 3). Embedding tools (ie, the Cloud, REDCap [Research Electronic Data Capture] data capture, the learning management system) into the platform to gather information on participant access and progress toward completing intervention activities allowed the lifestyle coach to assess participant engagement and success (eg, achievement of weight and behavior goals) [11,41].

#### Creation of Interactive Health Education Content in RT Intervention

Once Y-Living health education lessons were updated for the rural context, the RT team developed the interactive version of the lessons. The design and delivery of the lesson content were tailored to address the learning needs and the cultural and community characteristics of low-income Latino families [6]. We used avatars resembling study participants (eg, Latino adults) to present content, modified activities to reduce numeracy and literacy levels, and used narration and graphics to enhance content comprehension and retention. We also selected activities to reflect community context, limited the amount of information on each screen to reduce overload, and only included information from trusted resources within the lesson [42,43]. Finally, we used gamification to increase motivation and engagement.

E-learning authoring software Articulate Rise [44] and Articulate Storyline [45] were used to create the health education content. Articulate Rise was relatively easy to use and was well-suited for rapid deployment; however, we learned that there were limited options for customizing the lesson content to be interactive and address intervention needs revealed in the RT pilot study. As a result, the RT team decided to use Articulate Storyline to enhance lesson customization and participants’ interactive learning experience. The RT team converted PowerPoint slides that were created for the IP intervention as a basis for lesson content to develop each of the 24 RT lessons (2 lessons/week). Lesson titles remained the same, as did the lesson topics. RT intervention lessons followed the same structure as those for the IP intervention whereby each began with a motivational or spiritual message (ie, “Higher Thought”), followed by a recap of the previous lesson, the objectives for the current lesson, a pretest, lesson content, a posttest, and finally, physical activity videos. After completing a lesson, the participant is prompted to evaluate the lesson content and provide feedback on what they “liked” or “didn’t like,” and comments that could be used for improving the lessons for future versions.

The RT team developed interactive activities within each lesson to increase participant’s knowledge and self-efficacy to change physical activity and diet behaviors. Examples of interactive skill-building activities include creation of SMART (ie, specific, measurable, adjustable, realistic, time-based) goals for physical activity and dietary intake, practice reading *nutrition facts* food labels to assist in selection of healthier choices when grocery shopping, and identifying healthier choices when ordering food at a restaurant. All lessons were narrated to increase participants’ engagement with the lessons. By narrating the lesson content, the amount of text that the participants had to read from the screen was greatly reduced, and the information could be provided in more depth. The intervention staff wrote and recorded narration scripts for each lesson.

To provide the RT intervention participants an experience similar to that of the IP participants, the RT team video recorded physical activity sessions and cooking demonstrations conducted by Y-Living program staff from the YMCA. These videotaped activities were similar to the ones used with participants in the Healthy Frio IP program. Videos included Zumba, salsa dancing, boot camp style-physical activity routines, 22 cardiovascular fitness routines, and demonstrations for healthy ways to cook proteins vegetables, fats, and grains. The RT version included additional curated videos that demonstrated various physical activity routines available on YouTube. The criteria for selecting the videos were that they were primarily suitable for beginners, did not require any equipment, included a variety of physical activities for both strength training and cardiorespiratory fitness (ie, yoga, stretching, body weight exercises), and were fun. Each lesson included 2 physical activity videos: 1 for adult participants, and 1 that was more suitable for children. Narrated instructions introduced each video.

#### Selection and Testing of Digital Technologies for Delivery of Healthy Frio RT Intervention

The RT team underwent a tedious process of testing, developing, and integrating various hardware and software tools that would be supportive and cost-efficient for delivery of the RT intervention. Major considerations included (1) ease of use with user-friendly interface and minimum data entry burden, (2) product reliability (hardware durability and signal strength), and (3) ability to collect information from the tools via web-based API [7,29]. Table 3 lists the hardware and software utilized for delivering the intervention with descriptions of functions. With the exception of the smartphone, all of the equipment is provided to participants for a 12-week intervention period. Following the completion of the intervention, participants return the tablet computer and the weight scale, but allowed to keep their Fitbits and receive a digital weight scale.

**Table 3:** Technology components and functions used by remote technology intervention.

Technology component			Function
	**Hardware**		
		Verizon Ellipsis 10 tablet computer with mobile Wi-Fi hotspot and data plan	Intervention content delivery device to access the interactive health lessons; gathers and uploads information from monitoring devices
		Fitbit Flex 2 activity tracker	Personal sensor to track physical activity and sleep
		Nokia Body digital scale	Bluetooth scale to track weight
		Smartphones (participants’ personal)	Device used to view SMS text messages from the study
	**Software**		
		MessageSpace	Automated SMS text messaging system to send motivational texts and reminders to participants
		Moodle Learning Management System	Delivery of intervention content; tracks participant viewing of program lessons
		Fitbit and Nokia Health Mate apps	Apps to help participants self-monitor physical activity, food intake, water consumption, sleep, and weight. Weight syncing through Nokia Health Mate app.
		Google Hangouts/Skype	Video conferencing apps to conduct group counseling calls with health educator
		MX Player	Video player to view physical activity videos
		Electronic games	Games related to physical activity and nutrition for child participants

**Figure 4:**
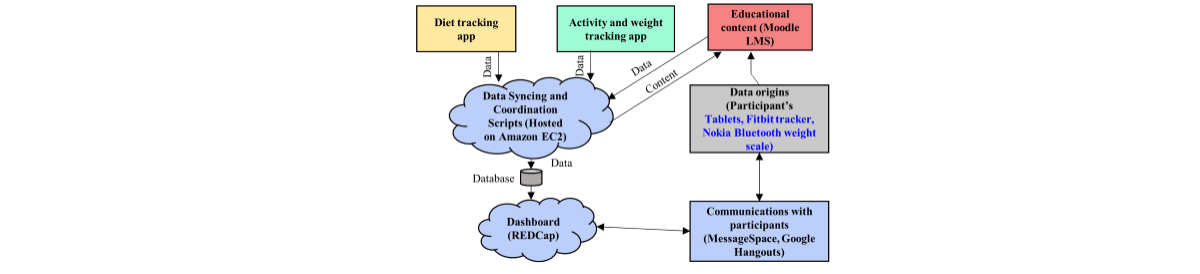
Healthy Frio remote technology intervention system architecture. EC2, Elastic Cloud Compute; LMS, learning management system; REDCap, Research Electronic Data Capture.

Figure 4 shows the system architecture constituting the selected digital technologies and their integration. Such architecture enables successful data collection and communication with participants during the RT intervention. The green components integrated in the system interface with Google Hangouts [46] or Skype [47] are for lifestyle coaching while collecting and processing data and communicating with the participants. These advanced messaging tools, when connected to REDCap [48], provide a communication channel to interact with participants. Data synchronization is typically done using a Bluetooth wireless connection with a smartphone or computer app. The participants can set their specific goals as reference marks and challenge themselves to achieve higher goals. Many of these apps allow tracker owners to connect with peer groups or friends, and some allow sharing data with other users. We chose not to use this function because the RT intervention is not group based. Some tools also have an option to connect to other third-party, variable-specific (eg, calorie counting or meal tracking app) tracking apps (eg, Fitbit [49]) that allow for tracking dietary intake. There exists significant value in observing diet trends over time and understanding the correlation between calorie expenditure and activity levels. During our evaluation study for activity trackers, we observed that Fitbit trackers offer the convenience of tracking users’ calorie intake, sleep duration, steps, miles, and activity minutes for the day.

To provide access to educational content during the intervention, we used Moodle as the learning management system to deliver program content, manage health education lessons, and track progress [50]. In other words, the lessons (content) created on Articulate Storyline were uploaded to Moodle for participants to access. Completed content is hosted on Amazon Elastic Cloud Compute (EC2) server [51] and is accessible to participants via a Moodle app on the tablets. Data from the aforementioned services are collected and retrieved in real time using corresponding manufacturer’s APIs (Fitbit, Moodle SQL database on EC2, and Nokia [52]) and reside in the database hosted on Amazon EC2 server. Data synchronization and coordination scripts take care of this process. Data are further postprocessed and are accessible to the lifestyle coach and intervention team on the Dashboard supported by REDCap. Alternatively, one can use Microsoft Azure [53] or Google Cloud [54] as the centralized server for incoming data from the components involved in the integrated digital health system. Table 4 presents lessons learned and design implications to address various design goals that are deemed necessary to engage participants and strengthen the human touch in the intervention.

**Table 4:** Summary of goals, design implications, and lessons learned in the design of remote technology intervention.

Design goal	Lesson learned	Design implication
To investigate whether usage of the tablet by parent and child affects user engagement and frequency of regular synchronizing of hardware devices with respective apps on the tablet.	Parents and children felt a burden with regard to synchronizing their activity, entering dietary data and weight, due to repeated log-in and logout from their respective accounts on the apps. This extra step led to very few, if any, syncs of their data.	Parents and children should be able to synchronize their data without the hassle of logging in and out. We created clones of the apps that are used by both parents and children, and their devices are configured accordingly to their cloned version and accounts. This improved data synchronizing and we are able to get frequent updates on their syncs.
To check the ease of access to educational content and health knowledge via Moodle, enhance access to information, get proper feedback for improving the content.	Navigating the Moodle app is challenging as there are many steps to take before landing on the course page to access lessons. This dissuaded participants from accessing weekly lessons. Due to poor network connectivity, downloading of lessons from the app is inhibited or is slow, further dissuading participants from accessing the lessons.	The app’s configuration settings on the Amazon EC2^a^ server as well as on the tablet should be changed. With the configuration changes, participants are able to directly see the list of lessons upon opening the Moodle app. Providing offline access to content (ie, via SD^b^ cards, download of complete 12 weeks of content from Moodle app onto the tablet) should resolve the network-related issue affecting access to lessons. This is essential when internet access is poor.
To investigate whether regular one-on-one communication can fill the gaps due to missing data collected remotely.	Due to diverse technical capabilities of participants in the intervention, they tend to forget some steps in constantly synchronizing the devices, accessing the content, and entering nutrition details.	SMS text messages as reminders, polling questions, surveys, and one-on-one video calls with health educator help remind participants about various aspects of the intervention, which in turn helps collect a more robust set of data.
To observe active logging of water consumption and tracking calories by entering dietary details and calories consumed.	Logging food and consumed calories requires manually entering or scanning the food item. Participants mostly either skip entering it or do it less often than required.	Regular SMS text messages as reminders and one-on-one video calls with Health Educator motivates and helps participants log food intake, keep track of their daily consumption, and stick to weight goals.
To evaluate tools and methods to enhance tablet usage with ease of navigation for regular synchronizing of data as well as for constant motivation and participant engagement.	Direct access to apps with little to no information to enter manually, easy methods of synchronizing their devices to the apps, automatic notifications from Fitbit (including device lights and vibrations), Health Mate, and Moodle apps seem to motivate participants to monitor their healthy living.	Tools for the intervention should be as integrated as closely as possible with the participants’ daily schedule. User-centered design will support end-user engagement, improve user’s experience, and encourage attentiveness toward the intervention.

^a^EC2: Elastic Cloud Compute.

^b^SD: secure digital.

#### Limitations of Using Commercial Products in Research: Access, Security, and Privacy

The potential for digital tools such as mobile apps and wearable sensors to enhance behavioral health is substantial. However, the use of tablets, wearable trackers, and other technologies for digital health interventions introduces additional complexity for participant’s data security and privacy [55]. One should carefully evaluate security issues, conforming to rules and regulations in the settings where these interventions are implemented [23]. Participants signed a consent form, which informs them about the heterogeneous RT environment and the type of data collected.

All the constituent apps in the Healthy Frio RT intervention have different security aspects. The current RT intervention delivery leverages Fitbit, Nokia Health Mate, and Moodle apps, which rely on remote servers for storing and processing the participant’s data, and adds to security challenges. Hence, the computer engineers and developers need to consistently secure servers, the transmission of data, and the involved software through their proper configuration. To address these issues, we performed a thorough evaluation of the complete RT intervention delivery infrastructure by observing the data and sensitive information flowing through multiple apps, modules that we developed leveraging corresponding software development kits, libraries, and APIs. This helps to anticipate potential security issues in order to take preventative measures. The third-party libraries and code have been assessed for any security vulnerabilities or other reported problems. Other safety practices include observing the differences between different APIs that enabled us to configure the apps properly for the security-related features and handle the permissions for data collection. This research helped us in adapting the code base accordingly to avoid compromising the security of participants’ information. We also generated participants’ credentials for the involved apps securely and ensured the passwords are not stored in the remote server in plaintext. We avoided storing their passwords and instead let them authorize our system to collect their activity, weight, and educational content access data prior to the start of the intervention. Finally, we used Amazon EC2 to deploy and maintain our remote server that communicates with the apps. While Amazon provides a secure environment including privacy protection, we took appropriate measures to configure the server for Fitbit, Nokia Health Mate, and Moodle course data-retrieval systems.

### Pilot Study

The prototype of the Healthy Frio RT intervention was tested with 5 parent–child dyads in a 3-week pilot, using content from the first 3 weeks of the intervention (ie, 6 lessons). The purposes were to (1) test the performance, management, and maintenance of digital equipment—especially with regard to connectivity and syncing of Fitbit trackers and weight scales; (2) obtain feedback about the design of the lessons; and (3) obtain feedback on the experience of engaging in a remotely delivered lifestyle modification program with digital technologies. Dyads were recruited from a convenience sample of eligible participants from the study population. The adult participants were all females aged 36 to 47. The ages of the children ranged from 7 to 14 years; 4 of the 5 families self-identified as Latino. Some of the participants had previously participated in an early IP intervention pilot. Therefore, they were able to provide feedback on the delivery of the RT intervention in comparison to the IP format.

The week prior to the start of the pilot study, participants came to an orientation session in which they were given an overview of the RT intervention, received their equipment as well as an instructional manual. The orientation session included a description of the 3-week pilot intervention, demonstrations of the use of equipment (eg, syncing Fitbit trackers and weight scales to tablets), introduction to apps installed on the tablet that would be used for self-monitoring, and a tutorial on accessing health education content on the tablet. RT pilot participants engaged in 3 focus group discussions in a community center (1 per week). Each family received a US $60 gift card for each focus group they attended. A focus group guide was developed prior to the pilot study that included questions related to the orientation session, lesson content and format, weekly SMS text messages received, use of equipment, and participants’ overall experience. Participants’ responses were transcribed in field notes which were then analyzed using content analysis [56]. RT team members decided to focus on manifesting content and develop categories using an inductive approach (ie, deriving categories directly from the text data). One member of the RT team was responsible for reviewing field notes, immersing herself in the data by reading through the notes several times. She then noted categories emerging from the data in an Excel file and shared them with other members of the RT team for verification. Several categories emerged from the data including hands-on training, user-friendliness (equipment), user-friendliness (apps), lesson content and aesthetics, and family engagement. These categories formed the basis for lessons learned from our pilot study.

### Hands-On Training

Participants stated that the orientation session needed more time and many wanted more one-on-one time with staff members to guide them to use the equipment. Participants wanted to spend more time going through the apps on the tablet and more demonstrations on how to log-in/log out of the apps for syncing the equipment—especially because 2 people were using the apps (parent–child dyad). Many participants suggested creating a hotline number that they could call for help troubleshooting any problems experienced.

### User-Friendliness (Equipment)

A majority of the participants stated that the tablet was not very user-friendly. For instance, many found the tablet bulky and difficult to carry around. Participants indicated that they preferred a smaller tablet that they could carry around with them (in their purse). However, participants also stated that they really liked using the tablet to view the exercise videos that were provided during the second part of each online lesson. Most participants stated that they wore the Fitbit all the time (except when charging) and like using it to keep track of their steps and sleep.

### User-Friendliness (Apps)

Participants expressed that there were “too many applications or things to do.” In particular, participants did not like having to log-in/log out of the apps, for instance, to sync their child’s data in addition to their own in the Fitbit app. Participants also had a hard time navigating within the Moodle app to open and view the interactive lessons. Participants also had difficulty downloading the lessons to view. This issue was likely related to their cellular data connection speed.

### Lesson Content and Aesthetics

Overall, the participants stated that they liked the lesson content, including the interface design, colors, and layout. Several participants requested SMS text message reminders to view the lessons because it was “easy to put off.”

### Family Engagement

Though participants were encouraged to view the lessons with their child, most participants viewed the lessons during the evening or on the weekends and did not include their child. However, they stated that they shared the relevant information with their child later.

### Lessons Learned

Based on the findings from our pilot study, we learned that all of the equipment should be presynced and apps ready to be used prior to the orientation session. For the pilot study, when the participants were given the equipment during the orientation session, staff had to help them sync their Fitbit activity trackers and scales to the tablet for both the parent and child. Because the trackers and scales connect with the tablet using Bluetooth, this proved to be challenging due to multiple devices being in close proximity to one another. It was especially difficult for less technologically savvy participants. Additionally, both the parent and the child were using the same Fitbit app on each tablet, requiring them to login and logout each time they wanted to sync their equipment. This issue became a problem that caused their data to sometimes get mixed up, especially with the weight data from the scales. “Too many devices in one space using Bluetooth to sync Fitbits and scales to tablets” as one participant reported. We solved this issue by creating clones of the Fitbit app so that two copies of the app could be installed on the tablet: 1 for the parent and 1 for the child.

Many participants expressed that they had issues downloading the lessons to view. This issue was likely related to their cellular data connection speed. Because the participants lived in rural, south Texas, their cellular connectivity was less than optimal. Having a weaker cellular signal resulted in significantly long times for each lesson to download to the tablet, and caused the videos that were embedded into each of the lessons to often not be able to play. To solve these issues, we embedded all of the videos within the lessons rather than stream the lessons from their original source (primarily YouTube). This required having to download all of the videos from YouTube, and then importing them into the lessons. To address the issue of the lengthy time for downloading the lessons, which was exacerbated once videos were embedded due to larger file size, all of the lessons were predownloaded to the tablet prior to the equipment being given to the participants. It should be noted that making these changes resulted in the amount of data to be stored on the tablet hard drive exceeding the available space (16 GB). Therefore, we installed a micro SD (secure digital) data card (32 GB) into each tablet.

By addressing issues participants had syncing their equipment to the tablet and viewing online lessons, we were able to address many of the challenges that occurred during the pilot. For instance, we were able to provide more time during the orientation session so participants have more hands-on training in using the equipment and apps and we enhanced the user-friendliness of the tablet, Fitbits, and apps.

## Conclusion

We designed a digital technology–based intervention to deliver a lifestyle modification program commonly delivered IP, by a lifestyle coach. The combination of digital technologies and live coaching affords the most efficient support to engage the study participants and close *the feedback loop* while providing constant exposure to the intervention content [29]. Digital health interventions are promising, but sometimes demonstrate mixed effectiveness [6,57], often due to low participant engagement and high study attrition [27,57] and a lack of integration of BCTs into interventions [32]. To address these concerns, we designed the Healthy Frio RT intervention by considering both the behavioral and psychological theories as well as systems engineering models [31,58]. The latter was critical in formulating technologies for efficient delivery and dynamic feedback in the RT intervention. We also incorporated key features identified in successful digital health interventions (eg, live lifestyle coaching) to enhance the efficacy of the RT intervention [28]. The RT development team was able to overcome many challenges through close collaboration of a multidisciplinary team consisting of behavioral health experts, computer scientists, an instruction specialist, and primary care providers. Finally, we embedded tools to collect information on participant adherence and engagement as part of the RT intervention. We hope that sharing our development process and lessons learned will help other researchers understand the factors influencing behavior changes in digital health interventions and guide the development of the next generation of digital health interventions.

## Abbreviations

API: application programming interface
BCT: behavior change techniques
EC2: Elastic Cloud Compute
IP: in-person
IT: information technology
mHealth: mobile health technology
PA: physical activity
PDS: persuasive design system
REDCap: Research Electronic Data Capture
RCT: randomized controlled trial
RT: remote technology
SD: secure digital
SQL: structured query language
